# Chemistry as a Branch of Medical Education

**Published:** 1853-07

**Authors:** 


### EDITORIAL DEPARTMENT.

*Chemistry as a branch of Medical Education.*—In the circular of every medical college which reaches us, we see chemistry set down as one of the branches of education, one of the chairs, as they call it. We have often speculated upon this matter and wondered if it were not a relic of past ages, a sort of superstitious observance which has come down to us from a remote antiquity, and which is kept up for the same reason that most persons vote for one side or the other at elections, because our fathers had done so before us.

A four months course of chemistry has something so preposterously ludicrous in the very enunciation of it, that we cannot think of it as a sober verity. To teach a set of ignorant young men chemistry in four months, is a harder task than to build Aladdin's wonderful palace in a single night. We would defy even the mighty Djinn of the lucky Arab boy to accomplish such a feat. We remember hearing the venerable Professor Silliman, of Yale college, (how many years ago we had rather not confess,) say that it was absurd to attempt to teach chemistry in the limited time allotted to him, (some nine months,) and that the whole course of instruction in this department would have to be changed, because the time allowed for its study would not be more than sufficient for the examination of one of its numerous departments. This was said, be it remembered, before the doctrine of organic radicals had been agitated, before the Protein question had been sprung upon chemists, before Liebig had become at all conspicuous, and while Lehmann was still playing marbles. How much more energetically then can we now repeat this dictum of the venerable chemist.

If we examine the method pursued by the professors of chemistry in the medical schools, we find, as far at least as our observation extends, that the old traditional plan is still adopted, a method, which, to say the least of it, is absurd, unnatural and useless. The professor harangues his class upon the physics of chemistry, wastes all the early portion of the session, while the students are still fresh, over electricity, galvanism, magnetism and the rest of the imponderables, laying down principles which might possibly be very useful to engineers, and which it is very desirable that medical students should learn, but which certainly should not be prosecuted, as they are, to the neglect of infinitely more important matters. After the Christmas vacation, the actual chemical course begins. Then we have the atomic theory, and the chemistry of the gases and the metalloids, following one another in the order laid down in the text-books, till the chemist comes to that portion of his course which really begins to be important, and then he finds that irrevocable time has gone and the approaching close of the session admonishes him to hasten. So he dashes whip and spur through the poisons, and through organic chemistry, and in haste and confusion bundles up his papers. This is the miserable abortion which is called a course of chemical instruction for medical schools. It is utterly worthless to the student, and he cannot be so humbugged as not to see it. Consequently the chemical lectures are the standing *bore* of the session. Materia medica is dry enough, but the necessity of it is manifest; therefore the infliction is patiently endured. Chemistry, however, as taught, contains nothing which appears to the student of any practical utility; he therefore neglects and contemns it.

Now this is certainly not the fault of the science, nor is it exclusively or even chiefly the reproach of the student. The whole difficulty lies in the misapprehension of his duty on the part of the professor. His time is limited. He knows that it is simply impossible to teach chemistry in the time allotted him. He knows that a full and satisfactory account of the imponderables alone, illustrated with a suitable number of experiments, would take up all his time. He knows that he must omit much which is of the last importance to the student, that his pretended toxicology and organic chemistry must be a humbug too transparent not to be seen through by the merest tyro present. He knows, if he reflects a moment, that the whole existing system is radically wrong.

What then is the manifest duty of medical schools? To abolish the chair of chemistry? By no means. We are fully and deeply sensible of the immense importance of chemistry to the physician, an importance which is daily on the increase, an importance which it is impossible to overrate. There is not a disease in which some chemical question may not arise, there is not a prescription which does not demand some chemical knowledge. This, it is the duty of the professor to impart to his students, and any misfortune, accruing from their ignorance, is justly chargeable to him, if, by carelessness, neglect, or an imperfect system of teaching, he has failed to impart this necessary information. The indication then is to reform, not to abolish, chemical teaching.

How is this reform to be accomplished? Easily. The chemical professor is not to make the absurd mistake of supposing that he is commissioned to teach the whole round of formal chemistry, from the title page to the *finis* of a regular treatise. He is not to content himself with a slovenly synopsis of a text-book, interspersed with trite experiments. He is, on the contrary, to make himself thoroughly acquainted with the wants of the student, and to adopt the best system for supplying them. He should make toxicology prominent in his course; he should teach the reactions of the various medicinal articles, their chemical adulterations and the methods of detecting them ; and, above all, he should give his students the most complete possible view of the present state of physiological chemistry. He should, in short, perceive that his is a chair of ***applied chemistry,*** as really as is any chair in a school of mines, or of arts and sciences. The professor of chemistry in a school of art, who should waste the time of his pupils in formal chemistry would be hooted out of his position. And yet this is exactly what is done in our medical schools, so that students are annually turned out, notoriously and confessedly ignorant of this invaluable science.

Even applied chemistry could not be taught on the present arrangement of lecture hours. The professors must learn that chemistry is fast becoming quite as important as anatomy to the rational physician, and they must allow more time to their teacher of this science. The students, also, must be forced, by the requirements of the final examination, to pay more attention than they are at present inclined to pay to the study of chemistry.

In this manner a desirable reform might be effected, and though the time allotted to the department, would, under any arrangement, be insufficient, still the course would be more complete than it is at present. A vast amount of rubbish would be thus thrown off the shoulders of the professor, and he would be enabled to devote all his energies to the actual duties of his chair. The student would constantly see the application to his future wants of the facts he is learning, and would consequently more carefully treasure them. This is a wonderful stimulus to attention. When the anatomist tells his class that the sartorius muscle is a guide to the femoral artery, and that if ever they want to tie that vessel, they must be well acquainted with the course of the muscle, and the other relations of the artery, how much more profound is the attention with which they listen to him, than that with which they hear of the muscles of the back, or the intricate relations of the Vidian nerve. Why? Because they appreciate the utility of the facts they are learning in the former instance, and do not see what possible advantage they can be to them in the latter.

So too with chemistry. If the professor of that science would show his class the application of facts, as he announces them, the same interest would be felt in his teachings, as is now manifested for the practical facts of anatomy. The professor, for example, who should simply exhibit to his students the reaction of lime with oxalic acid, without giving them any idea of the use they might make of this valuable fact, would produce very little impression upon their minds. If, however, he were to give them a description of oxalic acid poisoning, and show them how often it takes place, and under what circumstances mistakes in reference to this acid were likely to occur, he would fix the fact on their minds, by showing them its use.

It is upon this basis that chemical teaching should be placed. Formal chemistry to a medical student is a shadow without substance. Applied chemistry is what he ought to be taught.

